# DNA Methylation Mediates Persistent Epileptiform Activity *In Vitro* and *In Vivo*

**DOI:** 10.1371/journal.pone.0076299

**Published:** 2013-10-02

**Authors:** Ziv M. Machnes, Tony C. T. Huang, Philip K. Y. Chang, Raminder Gill, Nicholas Reist, Gabriella Dezsi, Ezgi Ozturk, Francois Charron, Terence J. O’Brien, Nigel C. Jones, R. Anne McKinney, Moshe Szyf

**Affiliations:** 1 Department of Pharmacology and Therapeutics McGill University, McGill University, Montreal, Quebec, Canada; 2 Department of Medicine (Royal Melbourne Hospital), Melbourne Brain Centre, University of Melbourne, Parkville, Victoria, Australia; CEA - Institut de Genomique, France

## Abstract

Epilepsy is a chronic brain disorder involving recurring seizures often precipitated by an earlier neuronal insult. The mechanisms that link the transient neuronal insult to the lasting state of epilepsy are unknown. Here we tested the possible role of DNA methylation in mediating long-term induction of epileptiform activity by transient kainic acid exposure using *in vitro* and *in vivo* rodent models. We analyzed changes in the *gria2* gene, which encodes for the GluA2 subunit of the ionotropic glutamate, alpha-amino-3-hydroxy-5-methyl-4-isoxazole proprionic acid receptor and is well documented to play a role in epilepsy. We show that kainic acid exposure for two hours to mouse hippocampal slices triggers methylation of a 5’ regulatory region of the *gria2* gene. Increase in methylation persists one week after removal of the drug, with concurrent suppression of *gria2* mRNA expression levels. The degree of kainic acid-induced hypermethylation of *gria2* 5’ region varies between individual slices and correlates with the changes in excitability induced by kainic acid. In a rat *in vivo* model of post kainic acid-induced epilepsy, we show similar hypermethylation of the 5’ region of *gria2*. Inter-individual variations in *gria2* methylation, correlate with the frequency and intensity of seizures among epileptic rats. Luciferase reporter assays support a regulatory role for methylation of *gria2* 5’ region. Inhibition of DNA methylation by RG108 blocked kainic acid-induced hypermethylation of *gria2* 5’ region in hippocampal slice cultures and bursting activity. Our results suggest that DNA methylation of such genes as *gria2* mediates persistent epileptiform activity and inter-individual differences in the epileptic response to neuronal insult and that pharmacological agents that block DNA methylation inhibit epileptiform activity raising the prospect of DNA methylation inhibitors in epilepsy therapeutics.

## Introduction

Epigenetic mechanisms are known to maintain long-lasting gene expression programs. These mechanisms involve several levels of regulation, including chemical modification of the DNA molecule by adding methyl groups at specific positions, often involving the dinucleotide sequence CpG [1]. Such modification regulates the binding of the different transcription regulators, both enhancers and repressors, and the transcription machinery to control the expression of specific genes [2-4]. Recent data supports the hypothesis that differential DNA methylation patterns can form in response to experiences after birth [5], can be long lasting, and can affect brain-related phenotypes in both rodents and humans [6]. Furthermore, it has been previously shown, that inhibition of DNA methyl transferases (DNMTs) could affect excitatory neurotransmission in the hippocampus [7,8]. These mechanisms may explain the persistence of acquired epilepsy long after the original trigger has receded and account for inter-individual variations in development of epilepsy, in addition to or in the absence of genetic heterogeneity.

Several lines of evidence are consistent with the hypothesis that epilepsy might be mediated by epigenetic processes [9-12]. A popular antiepileptic drug, valproic acid, is a histone-deacetylase inhibitor [13] that induces DNA demethylation in cultured cells [14,15] and in the brain [16]. Furthermore, recent analysis of hippocampi from mice acutely treated with the chemo-convulsant, kainic acid (KA) demonstrated widespread changes in DNA methylation [17]. To test whether DNA methylation plays a causal role in epileptogenesis, however, it is important to determine whether genes critical for epileptogenesis are regulated by DNA methylation in response to a transient initial insult and whether these DNA methylation changes are essential for epileptogenesis.

In this study we tested this hypothesis by examining the changes in DNA methylation in the upstream regulatory regions of *gria2*, the gene coding the GluA2 subunit of the AMPA receptor. Evidence indicates that the presence of GluA2 subunit (encoded by *gria2*) in the heteromeric AMPA receptors impermeabilizes it to calcium [18,19], preventing possible calcium-mediated toxicity. Early and lasting downregulation of *gria2* expression observed in epilepsy models suggest that it plays a critical role in initiating the epileptogenic cascade, maintaining neuronal hyperexcitability [19,20] and is critical for the pathophysiology of mesial temporal lobe epilepsy (MTLE), the most common form of epilepsy acquired in adulthood [21]. Furthermore, knockdown of *gria2* in young rats resulted in seizure-like behavior and neurodegeneration [22]. The molecular mechanism mediating this phenomenon remains unclear, but epigenetic changes such as REST targeted regulation of *gria2* expression by histone hypoacetylation in response to KA treatment [23], have been implicated.

In this study we used a gene-targeted approach by monitoring methylation at specific CpG sites in *gria2*, a gene implicated by several lines of data in epileptogenesis, in an *in vitro* mouse and an *in vivo* rat model of epileptogenesis triggered by KA. We hypothesize that this change in methylation is persistent and that inter-individual variation in *gria2* methylation is associated with differences in epileptic bursting activity *in vitro* and the severity of epilepsy developed in an *in vivo* model. We then tested whether these methylation events functionally down-regulated the *gria2* promoter activity and whether the non-nucleoside DNA methyltransferase inhibitor N-Phthalyl-L-tryptophan (RG108) [24] blocked methylation of *gria2* and epileptogenic bursting.

## Results

### Epileptiform bursts triggered by KA in the hippocampus are associated with inter-individual variability of immediate and persistent changes in a DNA methylation of a 5’ regulatory region of the gria2 gene.

**Figure 1:**
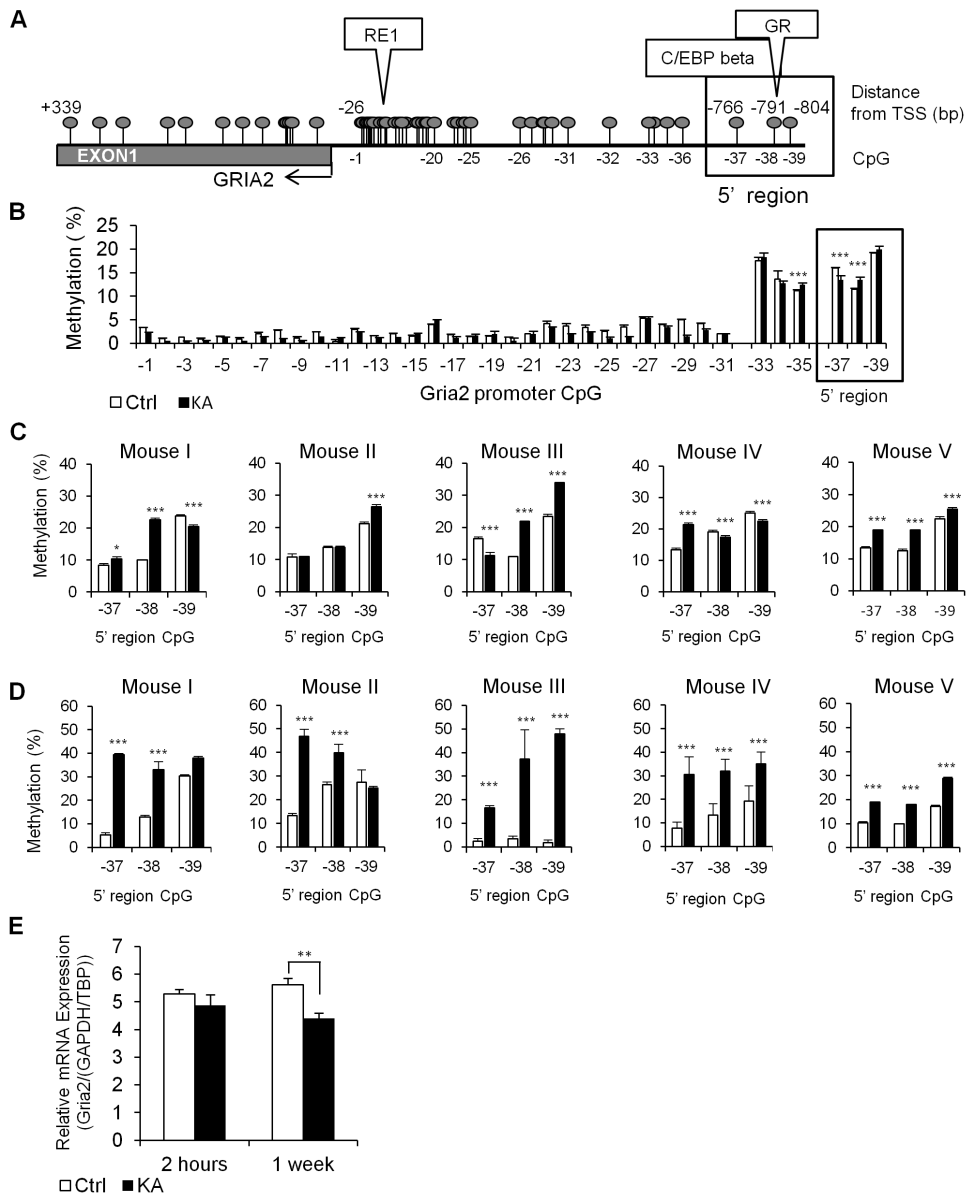
Methylation changes in *gria2* 5’ region in response to KA induced epileptiform activity. (A) Physical map of the *gria2* 5’ regulatory region and promoter region. CpG sites are marked by balloons and transcription factor predictions in the analyzed 5’ region are indicated above the physical map. (B) State of methylation of CpG sites in the proximal promoter of the *gria2* and the 5’ region in control and KA treated hippocampal slices (n=3 technical replicates); CpG 32 and 34 were not analyzed due to sequence restrictions. (C) Methylation differences between control and KA treated slices derived from the same mouse (n=4 technical replicates for each individual mouse) immediately after KA treatment for two hours, and (D) 1 week after removal of the drug (n=4 technical replicates, SD indicate technical errors). Inter-individual differences are apparent between littermate mice (Mouse I, II, III) and between mice from different litters (mouse IV, V) (E) *Gria2* mRNA expression levels in control and KA treated slices as measured by qPCR immediately after KA treatment (2 hours, n=5) and 1 week after removal of the drug (1 week, n=9). *Gria2* mRNA levels were normalized to TBP/GAPDH expression based on NormFinder. * p<0.05 ** p<0.01 *** p<0.005 as determined by Mann-Whitney U-test.

KA treatment of mature organotypic cultured hippocampus slices is a well-established *in vitro* model for inducing epileptiform activity [25]. Using this model we examined the state of DNA methylation of the proximal promoter of the *gria2* gene (Figure 1A) as well as a second region upstream to the proximal promoter (boxed; -766--804) in hippocampal slices after 2 hours of treatment with KA compared to drug-free cultured slices using pyrosequencing (Figure 1B).

The proximal promoter was generally unmethylated in all CpG sites (<5%) in both KA treated slices and controls with minute changes in DNA methylation between KA and controls (Figure 1B). However, a 5’ region positioned at -595 to -804 upstream of the transcription start site showed measurable levels of methylation and small but nevertheless significant differences in methylation between KA and control (CpG sites 35-39).

The 5’ region (-766-804) that exhibited consistent changes in DNA methylation with KA treatment in slices from individual mice (boxed in Figure 1A and 1B, CpG sites 37-39) was analyzed *in silico* using TRANSFAC [26]. The analysis identified several transcription factor binding sites, including CCAAT/enhancer-binding protein beta (C/EBP beta) and the Glucocorticoid Receptor (GR), which was previously shown to localize in Gria2 positive cells in the hippocampus and this colocalization was affected by epilepsy [27,28].

These results represent DNA methylation levels in a pool of 5 slices from different mice at the end of two hours KA treatment. However, it is well known that there are inter-individual differences in both animals and humans in the liability to developing epilepsy in response to a single brain insult [29]. A plausible hypothesis is that variations in DNA methylation of critical genes in the brain are associated with these differences. Genetically homogenous inbred strains are an ideal system to test this hypothesis. We first tested whether there are differences in the DNA methylation state of the KA responsive 5’ region in the *gria2* promoter between five individual mice. The results depicted in Figure 1C (see mice I, II, III) demonstrate variability in the basal state of methylation of the three CpG sites in this region between slices derived from littermate mice (the technical variability in measurement of the same mouse is indicated by the standard error), in addition to the differences observed between slices derived from mice of different litters (Figure 1C mice IV and V). In certain cases where an individual CpG site showed high methylation levels in the basal state (e.g CpG 39 in mice I and IV), we observed decreased methylation after the 2 hour KA treatment. This observation further highlights the intriguing finding that the DNA methylation response 2 hours after exposure to KA is different between slices from littermates.

We then determined whether the *gria2* gene would remain hypermethylated after KA was removed, serving as a “memory” of a transient exposure to the epileptiform inducer. We examined the DNA methylation state of slices that were treated with KA for 2 hours and were then maintained in standard culture media for one week in absence of the agonist and compared it to untreated controls. The results presented in Figure 1D show that transiently treated slices exhibited enduring increases in methylation of all three CpGs in the 5’ region (Ctrl 6.8±2.7, 15.1±5.36, 20.7±.8 KA 35.5±7.5, 36.6±2.2, 36.2±5.6 respectively) and a 2.4 fold in the average DNA methylation in the whole 5’ region of the *gria2* promoter (Figure 1A boxed) over control cultures (n=5, p=0.002). The difference in DNA methylation between treated and control slices after 1 week of incubation in drug free medium was higher than immediately after exposure to KA for 2 hours (Figure 1C). Importantly, each of the 3 CpGs sites in this region was more methylated in the treatment cultures than in control cultures (Figure 1D). Inter-individual differences between slices derived from the same littermate mice in the basal DNA methylation state and their response to 2 hours transient KA exposure were amplified following 1 week incubation in absence of the drug (Figure 1D).

We therefore tested whether the increase in DNA methylation in response to KA is associated with reduced expression of *gria2* mRNA. In order to accurately evaluate the levels of *gria2* mRNA levels we used NormFinder analysis to test possible reference genes which have previously been reported to be stable under seizure conditions (Glyceraldehyde-3-phosphate-dehydrogenase - *gapdh*, TATA binding protein -*tbp*, Hypoxanthine phosphoribosyl-transferase - *hprt1*, Neuron specific enolase *nse1*) [30,31]. We found that all four genes displayed a variability level well below the standard cutoff value previously established of 0.15 (Figure S1), indicating the value of any one of these genes as an accurate single reference gene. In order to increase the accuracy of our measurement, we used the combination of *gapdh* and *tbp* which was indicated by NormFinder to be the most stable. We found high variability in the *gria2* mRNA expression levels (n=5 cultures per condition) immediately after the 2 hour treatment in the KA-treated slices compared to controls, similar to the methylation response. The average expression of *gria2* mRNA in the transiently treated slices that were incubated in drug free medium for 1 week was 21.5% lower than in control slices (p=0.006) (Figure 1E), and showed variable levels of reduction.

**Figure 2:**
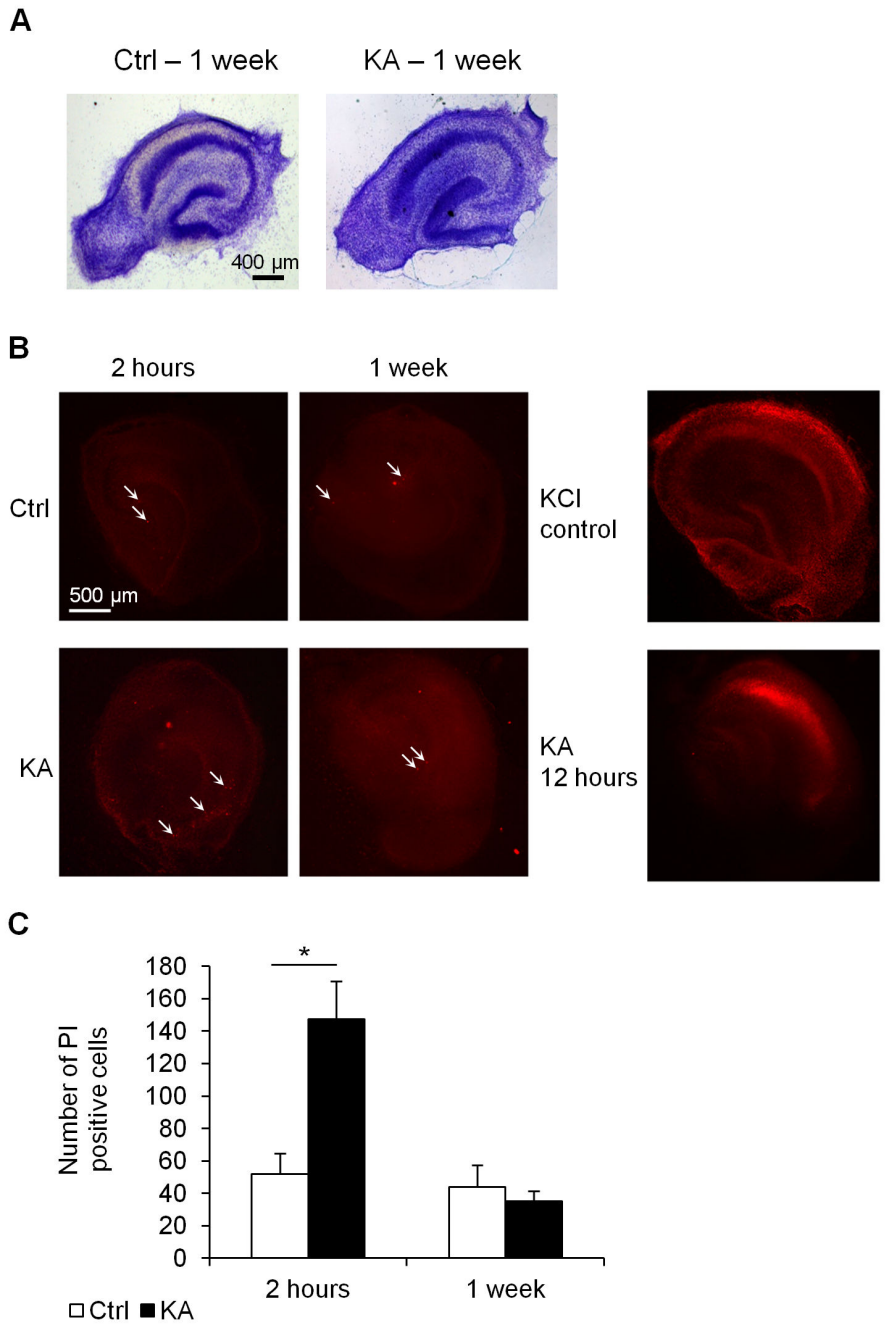
Cell death in hippocampus organotypic culture slices after KA treatment. (A) Nissl staining of mature hippocampus slices displaying normal neuronal organization in both control and KA treated slices 1 week after removal of the drug. (B) Propidium Iodine (PI) staining of the cultures at 2 hours treatment with KA (KA 2h), control (Ctrl 2h) and 1 week after removal of KA (Ctrl 1 week, KA 1 week) to evaluate cell death. 12h KA (KA 12 hours) was used as a positive control for levels of cell death reported in other KA models and 15.7M KCl was used as PI staining positive control (KCl control). PI positive cells are labeled in red. White arrows point at sample PI positive cells (C) Quantification of total number of PI positive cells in the different conditions show a small but significant increase in the number of positive cells immediately after 2h KA treatment compared to control, and no significant change after 1 week recovery (n=4). * p<0.05.

**Figure 3:**
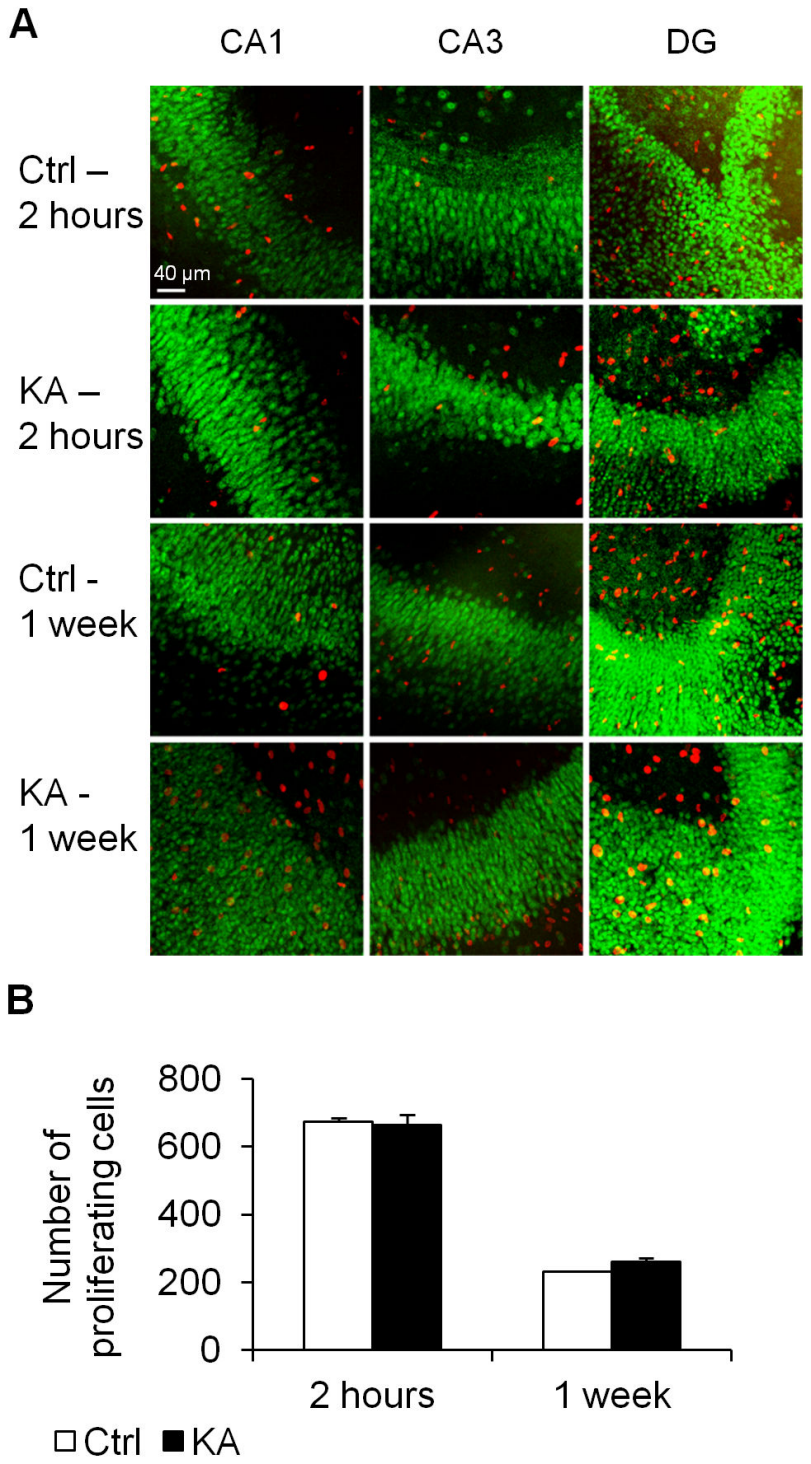
BrdU staining for visualization of proliferating cells after KA treatment. (A) BrdU incorporation [30] in the different regions of hippocampus slice cultures (CA1, CA3 and dentate gyrus – DG) after 2 hour treatment (Ctrl 2h, KA 2h) and 1 week recovery (Ctrl -1 week, KA -1 week). Neuronal cells (NeuN positive) are stained green (B) Quantification of the total number of proliferating cells (BrdU positive) show no significant difference between KA treated slices and control immediately after 2 hour treatment, or after 1 week of recovery (n=5).

For each allele, the methylation profile of a specific site is either methylated or not methylated, with each cell contributing an extreme of either zero or one hundred percent methylation to the cumulative measurement (i.e. percent of methylation measured gives an indication of the percentage of cells that are methylated in the slice). We considered an important set of potential confounders namely that the DNA methylation changes observed could have been caused by either DNA synthesis or cell death. These are especially important considering that the hippocampus is a heterogenic tissue and that changes in *gria2* levels have been previously associated with neuronal cell death associated with epileptic injuries [32]. Here we used several methods to evaluate the physiological condition of our slices. First, we applied Nissl staining (Figure 2A) to examine the structural integrity of the hippocampal slices. No swelling or vacuolization of cells, typically seen during cell death or apoptosis, was observed suggesting that the integrity of the hippocampus was not affected by 2 hours treatment with KA. Secondly, propidium iodine (PI) staining was performed to test for cell death by necrosis. We evaluated cell death induced by exposure to KA for 12h and found severe cell death throughout the slice similar to previous reports [33,34]. A positive control for the PI staining was also conducted by incubating a slice in high KCl solution prior to the staining (Figure 2B). A small amount of PI positive cells was observed in both the control and treated slices (Ctrl 52.0±12.78 KA 147.5±23.40 p=0.025) immediately after the treatment and an even smaller number 1 week post-treatment (Ctrl 43.8±13.72 KA 35.25±6.02 p=0.619; Figure 2C). We found a significant increase in PI positive cells after the 2 hour KA treatment. This difference correlates to less than 2% of the overall number of cells (9266.7±1430.4 as evaluated by manual counting of dissociated neurons in a Neubauer-Improved cell counting chamber). Thirdly, we used BrdU incorporation to evaluate cell proliferation in slices of both treated and untreated samples (Figure 3A). There was no significant difference in the total number of proliferating cells between the treatment and control samples either immediately after the KA treatment (Ctrl 672.5±11.79, KA 663.7±30.78, p=0.799) or after 1 week of recovery (Ctrl: 230.5±2.23, KA 260.0±12.39, p=0.290; Figure 3B). These minor differences between the number of control and KA treated dying and proliferating cells suggest that the persistent DNA methylation changes in response to KA treatment must represent a much larger percentage of cells that changed DNA methylation than the small number of dying or proliferating cells.

In summary, inter-individual DNA methylation differences in this region of the *gria2* promoter exist in otherwise genetically identical mice. Most importantly, there are differences in the responsivity of the DNA methylation state of these CpG sites to KA insult and these differences are enhanced during the drug-free incubation period creating large differences in the long-term DNA methylation state between different individuals with a “history” of transient exposure to KA insult. The response of the DNA methylation state does not seem to reflect a variation in cell death or proliferation.

### Variations in DNA Methylation associate with differences in bursting activity

**Figure 4:**
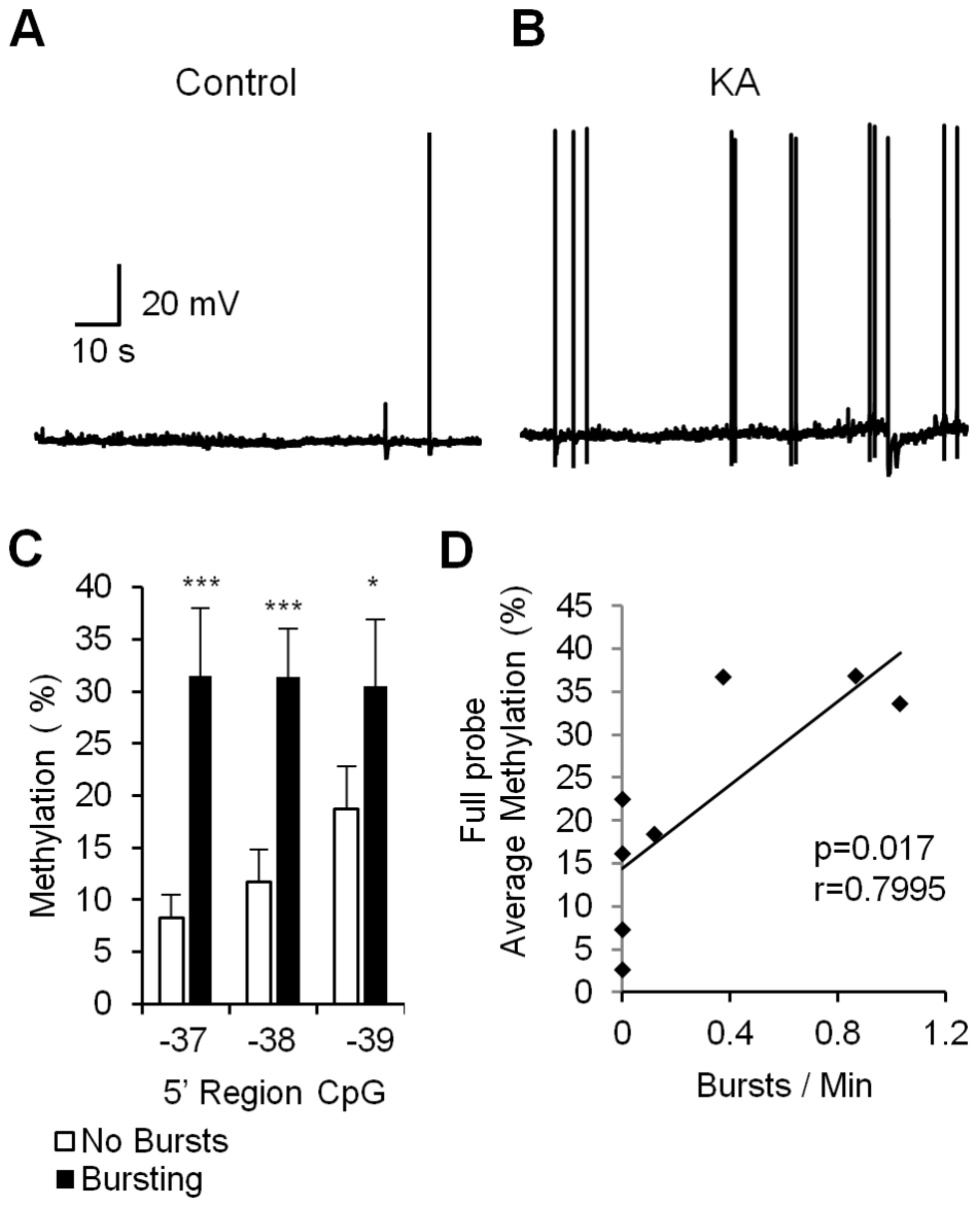
Correlation between epileptiform burst activity and *gria2* 5’ region CpG methylation levels 1 week after removal of drug. (A) Sample neurons’ membrane potentials recording in current-clamp mode of Control hippocampal slices and (B) 2 hours KA treated slices 1 week after removal of the drug and incubation in drug free medium. Three or more spikes were grouped into bursts if the inter-spike interval was smaller than 600 ms. (C) Average methylation levels of *gria2* 5’ region CpGs in either slices that exhibit spontaneous bursting and those that don’t exhibit bursting 1 week after 2 hours exposure to KA (n=4). (D) Correlation between bursting frequency and average probe methylation levels (p=0.017, Pearson’s r=0.7995). *p<0.05 ***p<0.005.

The inter-individual differences in DNA methylation as observed in the *gria2* promoter region beg the question of whether variations in DNA methylation in response to KA are associated with differences in long-term electrophysiological activity of the hippocampal slices. We therefore conducted membrane potential recording in single-cell current-clamp mode of control and KA-treated slices after one-week recovery (Figure 4A,B). Spikes were detected offline and 3 or more spikes were grouped into bursts if the inter-spike interval was smaller than 600 ms. We found that 10 out of 19 slices exhibited spontaneous bursting activity of the measured pyramidal neurons after the KA treatment, with an average of 6.21±3.39 bursts per slice (64.79±30.26 spikes per slice). Two control slices out of 16 did have some bursting activity, with an average of 0.25±0.14 bursts (3.56±2.11 spikes per slice, p=0.029). We then compared the DNA methylation level at the *gria2* 5’ promoter region in slices that exhibited bursting (n=4) versus non-bursting slices (n=4) (Figure 4C). DNA methylation levels were determined in four technical replicates for each slice from the same mouse.

Significant hypermethylation was observed in all CpGs in the examined region of *gria2* in the bursting slices relative to the non-bursting slices (p<0.001, p<0.001, p=0.017 respectively). The difference in average DNA methylation of the 3 CpGs in this region was highly significant as well (p<0.001).

We then correlated bursting and DNA methylation in this *gria2* 5’ region across all individual hippocampal slices. This analysis provided compelling evidence for a correlation between burst frequency and DNA methylation levels at the examined *gria2* promoter region (Pearson correlation p=0.017 r=0.7995; Figure 4D).

### DNA Methylation of the gria2 promoter in a rat in vivo model of chronic epilepsy

**Figure 5:**
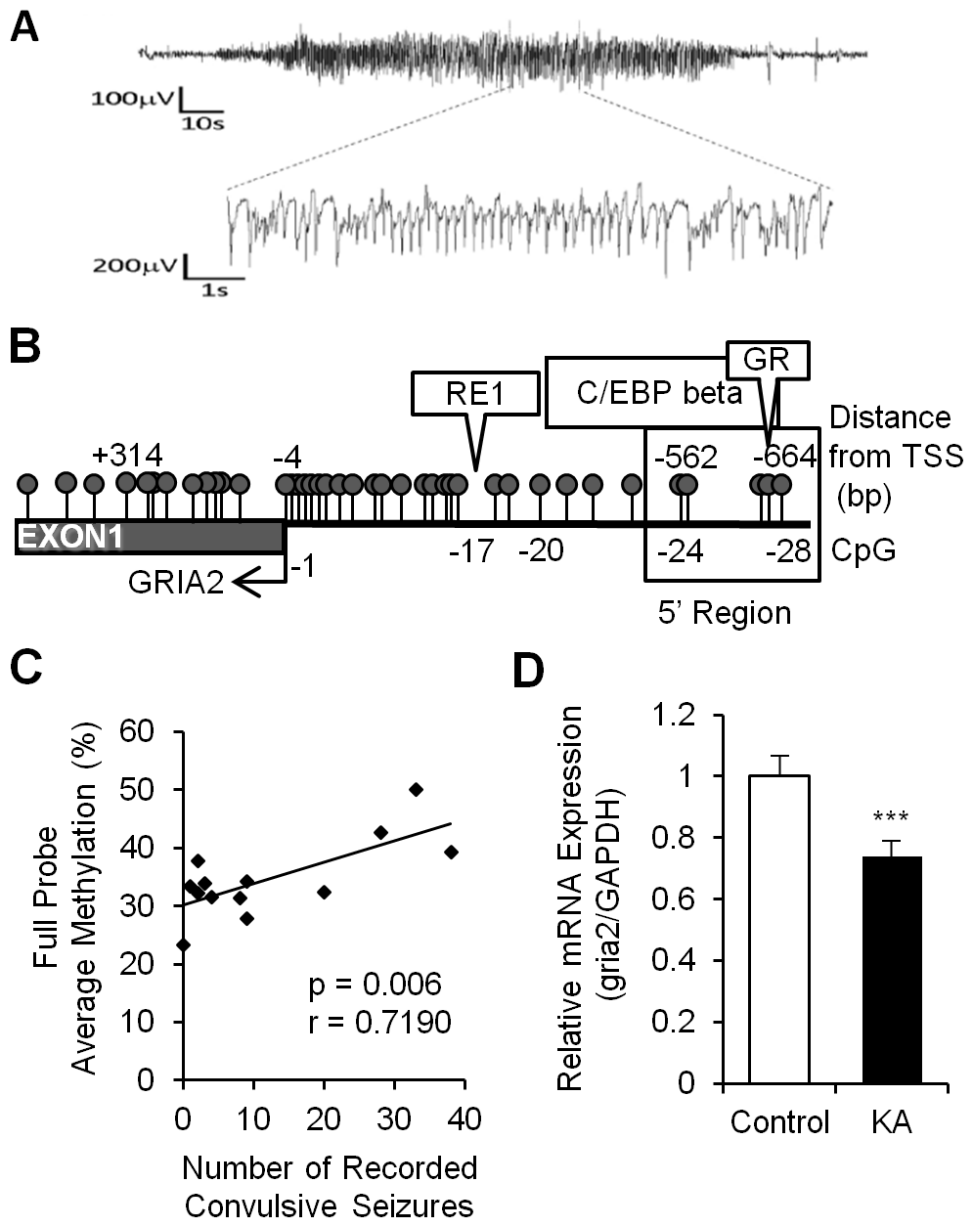
DNA Methylation changes in rat *gria2* gene promoter 5 ‘ **region in epileptic and control rats and their correlation with seizures**. (A) Representative electrographic recording of a seizure with synchronous video using Compumedics software. (B) Physical map of the rat *gria2* promoter (+320 - -664). CpG sites are marked by balloons and predicted transcription factors common to the mouse and rat 5’ region (-562--664) are indicated above the physical map. (C) Correlation between bursting frequency and average methylation levels of the *gria2* 5’ region (p=0.006, Pearson’s r=0.7190, n=13). (D) mRNA *gria2* expression levels in control and KA treated rats measured by qPCR 10 weeks after initial SE. ***p<0.005.

We then examined whether *gria2* DNA methylation behaves *in-vivo* similarly to what was observed *ex-vivo*. In order to assess this point, we induced Status Epilepticus (SE) in Wistar rats by intraperitoneal injection of KA. SE was terminated after four hours with diazepam. The animals were left to recover for ten weeks, at which point we performed a two-week period of video-EEG recording (Figure 5A) to record the frequency and severity of spontaneous seizures. Our results revealed high variability in the physiological response of the individual rats to the KA treatment (Table 1) ranging from a total of 2 class 0 seizures to 111 seizures (out of which 33 were class IV and V). Collectively, these animals were obtained from 3 litters, but no association existed between severity of the epilepsy and the litter. This observation demonstrates that the severity of the epilepsy which develops from this insult (all animals become epileptic following KA-induced SE) varies between different individuals from the same litter similarly to what was observed with the mouse hippocampal slices in culture (Figures 1C and D and 4D). We used this model to address the following questions: First, would the *gria2* promoter in rat hippocampus become hypermethylated in response to KA-induced SE and second, whether there are inter-individual differences in DNA methylation that correlate with the frequency of seizures?

**Table 1:** Seizure number and severity in rat 10 weeks after KA induced SE.

	Seizure number and severity					
Animal no.	Class 0	Class 1	Class 2	Class 3	Class 4
1	23	0	4	0	9
2	2	0	0	0	0
3	8	0	4	0	3
4	11	0	3	0	4
5	15	0	5	2	2
6	1	0	0	0	1
7	10	0	2	1	7
8	7	0	4	0	2
9	16	0	9	2	5
10	37	0	4	1	26
11	48	0	4	0	35
12	17	0	25	3	18
13	51	2	24	1	32

We focused on a 5’ upstream region (Figure 5B) that was previously shown to regulate *gria2* in the rat [35] and contains binding sites for transcription factors found in the differentially methylated *gria2* 5’ region in the mouse (Figure 1A). DNA was isolated from whole hippocampi from the rats after completion of the video-EEG recording. Similar to the mouse *in vitro* model, we found very low methylation levels (<10%) and no significant differences throughout the promoter, in both the control and high seizing animals (data not shown), and high inter-individual differences in the methylation state of the *gria2* 5’ promoter region in different rats. The high convulsive seizure rats exhibited higher DNA methylation in all CpG sites in the *gria2* 5’ promoter region (Figure 5C). We determined whether inter-individual differences in seizure behaviour (recorded number of convulsive seizures class IV and V) correlated with these inter-individual differences in DNA methylation of the tested *gria2* region (n=13). Our results plotted in Figure 5C show that, similar to the *in vitro* results, there is a highly significant correlation between the state of DNA methylation of *gria2* in the hippocampus of individual epileptic rats and their seizure behaviour (p=0.006, Pearson’s r=0.72). Furthermore, we found a significant reduction of 26.3% (p<0.001; n=13) in mRNA expression levels of *gria2* in the same treated rats relative to the controls, 10 weeks after induction of SE with KA (Figure 5D), as have been reported previously in the literature, and as we found in the *in vitro* model. The low seizure rats expressed on average 11.5% higher levels of mRNA than the high seizure rats but the difference between the high and low seizure groups did not reach statistical significance due to high variability in expression in the group and reduced numbers once the treated group was subdivided to low and high seizures. It is important to note that, on review of the video-EEG, no animals experienced Class IV or V in the 24 hours immediately preceding cull, which suggests that the changes in methylation are not due to the acute effects of convulsive seizures.

### Gria2 promoter activity is modulated by the state of methylation of the 5’ region (-528--719)

**Figure 6:**
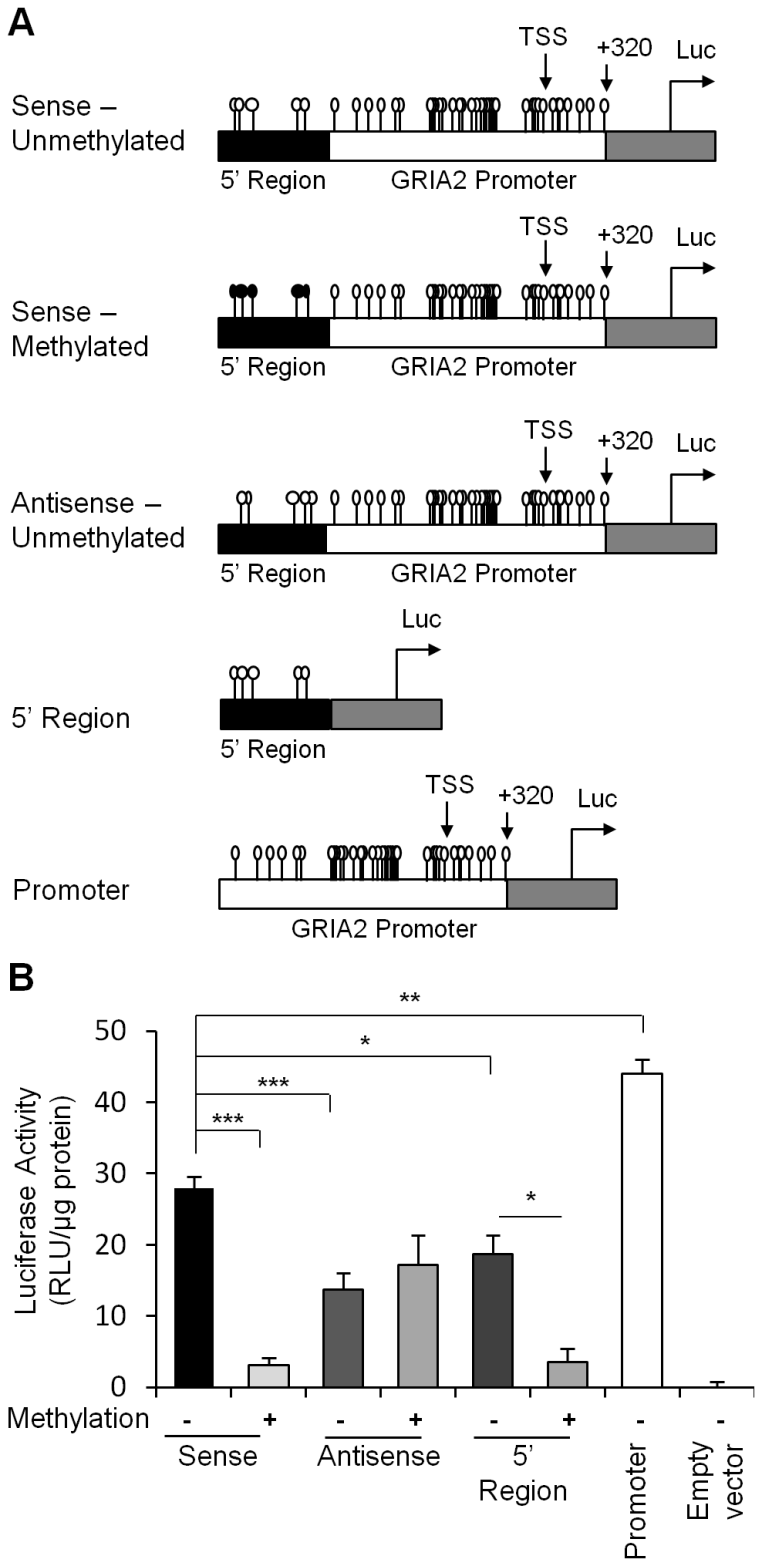
DNA methylation of the 5’ region silences the activity of the *gria2* promoter as determined by a transient transfection Luciferase reporter assay. (A) Physical map of the *gria2*-Luciferase reporter construct. The 5’ region CpGs in the probe were in-vitro methylated (black lollypop), or mock methylated (empty lollypop). The CpGs in the promoter region were left unmethylated (empty lollypop). Additional constructs were designed as controls; One containing the full promoter but with the 5’ region in a reverse direction (Antisense); second, containing only the 5’ region (5’ region); third containing only the unmethylated promoter (Promoter); forth containing plasmid without any promoter sequence (Empty vector). (B) The indicated constructs were transfected into SH-Sy5y (human neuronal cell line). 48h after transfection the cells were harvested, extracts were prepared and assayed for Luciferase activity and the values were normalized to total protein concentration. Results are average of (n=3) transfections +/- SEM. *p<0.05 **p<0.01 ***p<0.005 determined by a Student t test with Holm-Bonferroni correction.

The *gria2* promoter was previously found to contain several regulatory regions that interact with different transcription factors such as REST and NRF-1 [36]. We utilized transient transfection luciferase reporter assays to determine whether the *gria2* 5’ region (-528--719) that exhibited differential methylation in rats functionally regulated transcriptional activity and whether that activity was silenced by DNA methylation. We first generated a plasmid that contained the *gria2* 5’ region (-528--719) (Figure 6A) upstream to a Luciferase reporter gene in a pCpGL-Basic plasmid in either sense or antisense (reverse) direction (scheme in Figure 6A). The vector sequences were previously engineered to have no CpGs and are therefore not methylated by CpG methyltransferases [37], thus any effect of DNA methylation on expression of the reporter gene would be caused by the methylated CpG sites in the inserted test regions. We then subjected the plasmid to either *in vitro* methylation with the bacterial CpG methyltransferase *M.SssI* or to mock methylation. Following *in vitro* methylation of the *gria2* 5’ region we inserted by ligation the proximal unmethylated *gria2* promoter (+320 -528) in the sense orientation (Figure 6A) downstream to the methylated 5’ region. The state of methylation of this construct recapitulates the state of methylation of the *gria2* promoter *in vivo* in high convulsive rats. The ligated patch methylated or unmethylated constructs were directly transfected into SH-Sy5y (human neuronal cell line) cells without passing the plasmid through bacterial cloning to keep the state of methylation of the *gria2* 5’ region and unmethylated promoter region. Our results show that DNA methylation of the *gria2* 5’ region silenced luciferase activity when it was inserted in the sense orientation to the promoter but not in the antisense orientation (Figure 6B). Enhancer regions could direct activity in both orientations but promoters act only in the 5‘orientation to transcription start site. We therefore tested whether this 5’ region has independent promoter activity in absence of the proximal *gria2* promoter. Our results show that the 5‘ region of *gria2* could direct luciferase transcriptional activity independently and that this activity is silenced by methylation of its CpG sites. We further validated the presence of a transcript upstream to the known TSS and downstream to the *gria2* 5' region by RT- PCR using forward 5' primers residing on the 3’ edge of *gria2* 5'*-*region and reverse 3' primers around the known TSS. The amplified fragment was sequenced to verify that the fragment indeed represents mRNA upstream to the known TSS (see Figure S2 for physical map and sequence). Together, these results suggest that the *gria2* 5’ region (-528--719) that we found to be differentially methylated in response to KA treatment *in vivo* has promoter activity *in vitro* that is silenced by DNA methylation and that methylation of this region silences also the downstream unmethylated proximal *gria2* promoter.

### Inhibition of DNA methylation blocks epileptogenesis

Our results established that new DNA methylation events occur in response to KA treatment *in vitro*, that they could persist and amplify following removal of KA and they correlate with bursting electrical activity. The remaining crucial question is that of cause and effect; are the DNA methylation events a result of epileptogenesis and the changing cellular landscape in the brain [17], or do the DNA methylation changes triggered by the initial insult play a causal role in persistent bursting *in vitro* and seizures *in vivo*? We used a catalytic inhibitor of DNA methyltransferase RG108 to test whether DNA methylation activity is required for the development of bursting activity in hippocampal slices in response to KA.

**Figure 7:**
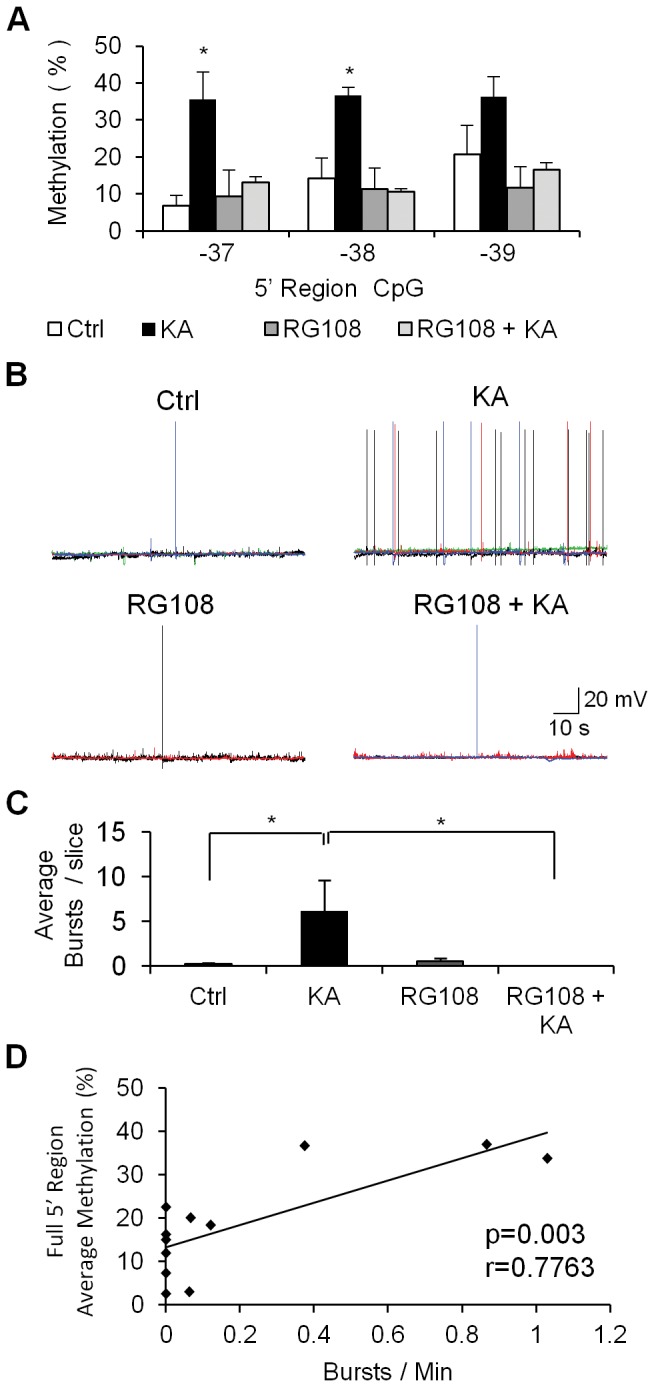
Effect of DNA methylation inhibitor RG108 on KA induced DNA methylation of the *gria2* 5’ region and epileptiform bursting 1 week following a transient 2 hour exposure. (A) Methylation differences between control (Ctrl, n=4 technical replicates on 3 different mice of origin), KA treated (KA, n=4 technical replicates on 3 slices from different individual mice), RG108 (RG108 n=4 technical replicates on slices from 2 different mice) and RG108 and KA treated slices (RG108+KA n=4 technical replicates on 2 different mice of origin) 1 week after removal of the drug and incubation in drug free medium. (B) Sample neurons’ membrane potentials recording in current-clamp mode of Ctrl, KA, RG108 and RG108 + KA treated samples. (C) Average spontaneous bursting activity of hippocampus slices after treatment with the different drugs. Significant increase in bursting activity can be observed in KA treated slices (n=19) vs. control (n=16). RG108 combined with KA treatment (RG108+KA, n=14) blocks the spontaneous bursting induces by KA treatment (n=19). Treatment with RG108 alone (n=12) does not have any significant effect on bursting compared to control slices (n=16). Significance between the different conditions in multiple-comparisons was calculated using Student t-test with Holm-Bonferroni correction for multiple comparisons. (D) Correlation between bursting frequency and average probe methylation levels in all the treatments samples (n=12) (Ctrl, KA, RG108 and RG108 + KA) (p=0.003, Pearson’s r=0.7763). * p<0.05 ** p<0.01 *** p<0.005.

Mature organotypic hippocampal slices were treated for 2 hours with either the DNA methylation inhibitor RG108 (100µM), KA (6µM), a combination of KA and RG108 or left untreated as controls. The slices were left to recover for one week without the drugs. The results presented in Figure 7A show that while KA treatment induced methylation changes as displayed by the hypermethylation of all the CpG sites in the *gria2* 5’ region, the DNA methylation inhibitor RG108 blocked this persistent increase in DNA methylation in response to KA. RG108 by itself had no effect on the state of DNA methylation. This suggests that there is no active demethylation or new DNA synthesis of this region in untreated slices that will necessitate the presence of DNA methylation activity to maintain the DNA methylation state. However, DNA methylation activity is required for KA induced DNA hypermethylation. We then determined whether blocking KA induced DNA methylation would also block bursting activity. As seen by the bursting frequency in Figure 7B,C, while RG108 had no significant effect on cultures that were not exposed to KA with an average of 0.58±0.34 bursts (6.25±3.69 spikes, n=12), it completely blocked bursting activity that is normally induced by KA in all of the slices (n=14) showing bursting activity (3.86±3.70 spikes per slice). The percentage of bursting slices were Ctrl 12.5%, KA 53%, RG108 25%, RG108 + KA 0%. When bursting activities and DNA methylation of the *gria2* 5’ region were correlated across samples of all conditions, there was a significant correlation between *gria2* DNA methylation and bursting activity (Figure 7D, p=0.003 Pearson’s r=0.7763). This data supports the conclusion that DNA methylation activity is required for the development of epileptogenesis in response to KA and is consistent with the hypothesis that increased DNA methylation of *gria2* 5’ region as well as other putative genes that were not measured in this study is involved in epileptogenesis.

## Discussion

Epilepsy can be triggered by either physical or neurochemical insults to the brain in humans and animals. The critical question is what are the mechanisms that mediate long-term consequences of these transient insults, such as chronic seizures? Another unresolved question is what are the mechanisms responsible for the inter-individual variation in the chronic response to a transient epileptoform insult? Human and animal epilepsy studies show changes in gene expression profiles [38-40], suggesting that mechanisms involved in epileptogenesis are registered in the genome and that there must be genomic mechanisms that mediate the long-lasting changes in gene expression in response to transient neural insult.

We used *in vitro* and *in vivo* rodent models of epileptogenesis triggered by transient KA exposure to test the plausibility that DNA methylation mechanisms are involved in long-term genomic memory of earlier neural insults. We focused on a single, well-established candidate gene, *gria2* as a plausible example, although we recognize that it is not exclusively targeted by KA and that several functional gene pathways are potentially involved in mediating the insult’s long-lasting effects [17]. Nevertheless, the study of DNA methylation alterations in this gene enabled us to establish the first principles of DNA methylation involvement in epileptogenesis. A causal role for *gria2* in epileptogenesis in young rats has been previously established. GluA2 encoded by *gria2* is the glutamate receptor subunit that inhibits Ca^2+^ permeability of AMPA/kainate receptors. GluA2 expression is deregulated in hippocampal regions after KA treatment [18,41]. Knockdown of *gria2* in hippocampi of young rats induced seizure-like behavior [22], supporting a causal role of *gria2* down-regulation in epileptogenesis.

Our study of *gria2* demonstrates that DNA methylation changes are triggered by KA using *in vivo* (Figure 5) and *in vitro* models of epileptogenesis (Figure 1) and that they last beyond the initial exposure *in vivo* (Figure 5) and *in vitro* (Figure 1) in two rodent species. Moreover, the long-term differences in DNA methylation between treatment and control are enhanced following a period of incubation in absence of the drug, suggesting a consolidation of the long-term response in the post-insult period. These results conform with the previously suggested hypothesis of the involvement of late-onset methylation-mediated gene silencing and epileptogenesis [42].

Our data is consistent with the hypothesis that DNA methylation of the *gria2* 5’ promoter region is involved in its regulation during epileptogenesis and that DNA methylation is required for epileptogenesis. First, the increase in methylation observed following one week of incubation in the absence of the drug (Figure 1D) is associated with a significant reduction in levels of *gria2* mRNA expression in the *in vitro* mouse model (Figure 1E) and the methylation levels of the *gria2* 5’ region in epileptic rats *in vivo* (Figure 5C) is associated with reduced expression of the mRNA (Figure 5D). Second, changes in DNA methylation and mRNA expression, which were measured in the heterogeneous cell population of the hippocampus were not due to proliferation or death of either glial or neuronal cells (Figures 2, 3). Third, the promoter activity of *gria2* is silenced in transient transfection reporter assays by the same methylation events that were shown to occur in the rat *in vivo* model (Figure 6B). Fourth, the level of *gria2* methylation after long-term drug-free incubation, following a single transient insult correlates with the amount of seizures measured *in vivo* by video-EEG (Figure 5), and with the levels of bursting *in vitro* measured by single-cell patch clamp recording of pyramidal neurons (Figure 4). Fifth, inhibition of DNA methylation, with a catalytic DNA methyltransferase inhibitor RG108 (Figure 7B,C) which causes inhibition of methylation of *gria2* results in parallel inhibition of bursting (Figure 7A). Moreover, the extent of change in *gria2* methylation in response to RG108 correlates with the reduction in bursting (Figure 7D). Although RG108 is a general DNA methylation inhibitor and most probably impacts the state of methylation of several genes in addition to *gria2*, considered altogether, these data along with previous publications demonstrating a causal role for *gria2* in epileptogenesis [18,22,41] are consistent with a critical role of DNA methylation in genes such as *gria2* in the long-lasting electrophysiological response to KA insult.

The fact that a DNA methylation inhibitor completely blocks long-term bursting in response to a transient KA treatment strongly supports DNA methylation involvement in epileptogenesis. Although we used a general DNA methylation inhibitor, which blocked increased DNA methylation of other genes as well, the results nevertheless provided evidence that the methylation of new sites in the genome is required for epileptogenesis. The possibility that DNA methylation mediates the long-term maintenance of an epileptogenic status points to new therapeutic directions in epilepsy. Further studies are required to delineate the genome-wide changes in DNA methylation affected by RG108 and their role in epileptogenesis, and the possible side effects of RG108 treatment on normal tissue. Interestingly, RG108 doesn’t alter the basal state of *gria2* methylation, suggesting that DNA methyltransferase activity is required for the KA-mediated changes in methylation, but its methylation state is not dynamically maintained in the normal state, since no cell division occurs in most of the hippocampus cells.

An excellent correlation was observed between the *gria2* methylation and both the bursting in the *in vitro* model and the seizures in the *in vivo* model, suggesting a quantitative relationship between DNA methylation of *gria2* and its electrophysiological function (Figure 4D, Figure 5C). A well-known enigma is why some individuals are more prone to developing epilepsy in response to an earlier brain trauma. The early identification of higher-risk subjects is of paramount importance when developing protective and preventive strategies. One plausible explanation for variances in the susceptibility to epilepsy is genetic differences. However, as differences in epileptogenesis appear among inbred, genetically homogenous mice and rats, an alternative hypothesis of a significant “epigenetic” contribution to this inter-individual variation should be considered. Remarkably, inter-individual differences in bursting in response to KA are seen not only in rats *in vivo* (where one could attribute variations in response to slight differences in experimental manipulation of animals), but also when brain slices from genetically homogeneous mice are placed under identical pharmacological, physical and chemical conditions *in vitro* and are provided an identical concentration of drug in solution in random Brownian motion. What we observed is that the initial relatively small DNA methylation response and the alterations that are consolidated following a long-term incubation period in absence of the drug, exhibited large inter-individual differences (Figure 1C, 1D and 4C). Importantly, these differences in methylation correlated with differences in bursting *in vitro* (Figure 4D) and seizures *in vivo* (Figure 5C). While we have to acknowledge that the averaged methylation differences for CpG -37 to -39 between KA treated and control slices are initially small, it becomes clear that these relatively small changes with high inter-individual variability are further amplified with time (Figure 1D). Even though we did not find any correlation between basal methylation level and the level of change immediately after treatment, or between the basal methylation levels and susceptibility for long-term bursting activity, these data provide strong support for the hypothesis that there is an inter-individual difference in the DNA methylation response to KA. This difference is at least partly responsible for the inter-individual differences in electrophysiological response and the chronicity of the epileptic state.

There is evidence that DNA methylation differences driven by experience and not exclusively by genetics also appear in humans. Notably, differences in DNA methylation were shown to emerge between monozygotic twins later in life, as well as during gestation [43-49]. These differences were associated with psychological disorders such as schizophrenia and bipolar disorder [50]. It is therefore plausible that as in rodents, variations in DNA methylation may play an important role in creating inter-individual differences in the emergence of epilepsy following brain trauma. If indeed DNA methylation mechanisms are involved, there are clear implications on diagnosis, prediction, prevention and therapeutics of brain injury and consequent epilepsy in humans. A critical question is whether some of the DNA methylation differences between individuals are present before the exposure to the insult and whether there are DNA methylation marks in peripheral tissue that could predict susceptibility to developing epilepsy either prior to or following a brain insult. Identification of such marks will have a profound impact on the treatment and prevention of epilepsy.

## Materials and Methods

### Animals

All animal handling procedures and experiments were approved by The Mcgill University Facility Animal Care Committee (FACC) in accordance with The Canadian Council on Animal Care (permit no. 5057) and the University of Melbourne Animal Ethics Committee in accordance with the Australian Code of Practice for the Care and Use of Animals for Scientific Purposes (permit no. 0911543). C57BL/6 mice were bred at the Faculty of Medicine animal facility at McGill University, Montreal. Male Wistar rats were inbred in the Department of Zoology, University of Melbourne Biological Research Facility (BRF), and housed from 5 weeks of age in the Department of Medicine (RMH) University of Melbourne BRF until the completion of the study. All facilities were under controlled temperature (20 °C) and lighting conditions (12 h light/dark cycle – lights on at 0600 h) and animals had *ad libitum* access to food and water.

### Mouse hippocampal Organotypic Hippocampal cultures

Mice were sacrificed at postnatal day 6 by cervical dislocation. The brains were rapidly excised, and the hippocampi microdissected out and sliced to 400µm in thickness by McIlvain tissue chopper. Each slice was mounted onto a poly-D-lysine-coated glass cover slide with chicken plasma (Cocalico, New Jersey) and thrombin (Sigma-Aldrich, St. Louis, MO), and inserted into a plastic tube containing 500µL media (25% heat-inactivated horse serum, 50% basal medium Eagle, 25% balanced salt solution; pH 7.4) for cultivation in a dry-air roller incubator at 36 degrees. Each organotypic slice was cultured for 3 weeks to allow differentiation and maturation of neural circuitries. The morphology of the resulting slices is similar to those observed *in vivo* [25]. To induce epileptiform activity, cultures were incubated with KA (6 µM) for two hours. Hippocampal slices were incubated with either 6 µM KA alone, 100µM RG108 alone (Sigma-Aldrich, St. Louis, MO), or RG108 + KA. Control cultures were incubated in fresh medium for the same duration of time. After 2 hours, the slices were either removed for immediate processing, or incubated in fresh drug-free medium for 1 week in the dry-air roller incubator. All culturing methods were carried out according to standards of the Canadian Council on Animal Care (CCAC)-approved protocols.

### Rat Post-KA Induced Status Epilepticus (SE)

The post-kainic acid induced SE rat model closely resembles the clinical features, ontogenesis, imaging and histopathological brain changes of human MTLE [51,52]. At 9-10 weeks of age, rats were exposed to 5 mg/kg KA [40] which induced SE, a period of sustained seizure activity. Four hours after the initiation of SE (defined as being unresponsive to external stimuli, and experiencing convulsive seizures), animals were given an injection of diazepam (5mg/kg ip) to cease the SE. Control animals received saline (0.9%, 2ml/kg ip) coupled with diazepam. Animals were then left to recover, and over the ensuing weeks, spontaneous recurrent seizures develop, leading to a diagnosis of epilepsy.

At 8 weeks post-SE, all rats were surgically implanted with extradural recording electrodes, as described in previous publications [53]. Briefly, rats were anaesthetized with isoflurane (5% induction, 2-3% maintenance) and a midline incision made on the scalp. The connective tissue was removed, and 6 burr holes were drilled into the skull. Brass recording electrodes were then gently screwed into the holes, and held in place by dental cement.

At 10 weeks post-SE, all animals underwent 2 weeks of continuous video-EEG recording (Compumedics, Australia) to record the frequency and severity of spontaneous seizures. At the completion of the 2 weeks recording, animals were rapidly decapitated, and the brains excised and immersed in ice-cold artificial CSF containing 125mM NaCl, 3mM KCl, 6mM MgCl_2_, 1mM CaCl_2_, 1.25mM NaH_2_PO_4_, 25mM NaHCO_3_, and 10.6mM glucose. The hippocampus structure was then microdissected using a dissecting microscope. One hippocampus from each animal was taken for RNA extraction, and the other one was snap-frozen for DNA extraction and pyrosequencing analysis of DNA methylation of *gria2* gene 5’ regulatory region.

### DNA Extraction

Whole hippocampus tissue was digested overnight with 400µg/ml Proteinase K (Roche, Germany) in Lysis Buffer consisting of 10mM Tris (pH 8.0), 400mM NaCl, 2mM EDTA, and 1% SDS (Sigma, St. Louis). The solutions were then saturated with NaCl and the homogenates were centrifuged. The supernatant was transferred to a new tube and was treated with 17 µg/ml RNAse A (Fermentas International, Canada) and was then subjected to phenol-chloroform extraction. The DNA was then precipitated out of the aqueous phase by adding 3 volumes of -80°C 95% ethanol and 20µg glycogen (Roche, Germany) as a carrier followed by high speed centrifugation. The resultant DNA pellet was washed in 700µl 75% EtOH, centrifuged again and the EtOH was air dried. The dry DNA pellet was then suspended in Tris-EDTA buffer (10mM Tris-HCl (pH 7.5) and 1mM EDTA (pH 8.0)).

### RNA Extraction

RNA was extracted from whole hippocampi using a Trizol reagent protocol (Invitrogen, Life Technologies, Carlsbad, CA) according to the manufacturer’s protocol. Extracted RNA was treated with DNAse (New England Biolabs) according to the manufacturer’s protocol. 500 ng of total RNA was used as template for cDNA synthesis using AMV reverse transcriptase (Roche Diagnostics, Laval, QC, Canada) and poly[12]_16_VN (IDT Technologies) as recommended by the manufacturer.

### Bisulfite Pyro Sequencing

Epigentek Bisulfite Kits (Qiagen) were used for bisulfite conversion of DNA as described in the manufacturer’s manual. 1µg of genomic DNA was used for a single-conversion reaction. Samples were prepared by performing nested PCR with one of the nested primers carrying a 5’ biotin modification. Primers used are listed in Table S1.

All primers designed against bisulfite-converted DNA using either MethPrimer online software [54] or pyromark assay design software (Qiagen) were synthesized by IDT Technologies. PCR reactions were conducted using Taq Polymerase (Fermentas International, Canada). Reaction conditions consisted of initial denaturation/enzyme activation at 95°C for 3 min, then 40 cycles of 95°C for 30 sec, annealing for 30 seconds at the temperatures listed below for the different primers, 72 °C extension for 30 seconds, and completed with a final extension step at 72 °C for 4 minutes. Annealing temperatures were: mouse outside PCR 54.8°C; mouse nested PCR 49.6°C; rat outside PCR 50.8°C; rat nested PCR 55.1°C. The nested 5’ primer contained a 5’ biotinylated nucleotide. Pyro Sequencing was then performed using a PyroMark Q24 machine (Qiagen) using the protocol described in the manufacturer’s manual. In brief, biotinylated PCR products were incubated with streptavidin sepharose beads (GE healthcare, Canada) for 15min in room temperature. Unbiotinylated strand was removed by denaturing with 0.2M NaOH, and the beads were washed with 10mM Tris (pH=7.5). Beads were released into 24 well plate containing 25µl annealing solution and 0.3mM sequencing primer per well. The plate was loaded onto the PyroMark Q24 machine using specific sequencing assay runs. The results were analyzed with PyroMark® Q24 Software (Qiagen).

### Real-time Quantitative PCR

Mouse mRNA expression levels were quantified by real-time quantitative PCR on a Roche LightCycler® 480 Real-Time PCR System using LightCycler480^®^ DNA SYBRGreen I master mix (Roche, Mannheim, Germany). Primers were purchased from IDT Technologies and are listed in Table S1. Amplification was performed using the following cycles: 10min at 95 °C followed by 45 cycles of 10 sec at 95 °C, 10 sec at 56 °C and 10sec at 72 °C. Amplification was followed by an extension for 10min at 72 °C and a melting curve cycle. Stability of the reference genes was determined using NormFinder (http://www.mdl.dk/publicationsnormfinder.htm). For rat *gria2* mRNA expression, Taqman Multiplex real-time PCR was carried out using an ABI prism 7000 sequence detector (Applied Biosystems, Warrington, UK), with 2 µL cDNA, 18 µm each primer, 5 µm probe, and Universal TaqMan 2 × PCR Mastermix (Applied Biosystems) to a final volume of 25 µL. All samples were run in triplicate. Primers and minor groove binder (MGB) TaqMan probes for *gria2* (Rn00568514_m1) and *gapdh* (Rn017775763) were designed by Applied Biosystems (Assay-on-Demand) to avoid genomic amplification. The thermal cycling conditions used during the PCR were: 2 min at 50 °C, 10 min at 95 °C, followed by 40 cycles at 95 °C for 15 s and 60 °C for 1 min. *gria2* mRNA levels were normalized to *gapdh* and the values were calculated relative to untreated controls.

### Nissl Staining

Organotypic hippocampal slice cultures were fixed in 0.1 M phosphate buffer (PB; pH 7.4) with 4% paraformaldehyde overnight at 4 °C. Slices were then washed several times and dehydrated for 10 minutes in 30%, 50%, 70% ethanol and then stained with 0.5% cresyl violet. Further dehydration was performed in 90% and 100% ethanol and 45 minutes in 100% xylene. Nissl-stained slices were mounted using Permount mounting medium (Fisher Scientific) and imaged with an upright Zeiss Axioplan 2 microscope equipped with a 2.5x objective and a Zeiss Axiocam high-resolution color digital camera (Carl Zeiss AG).

### In –Vitro Electrophysiological Recordings and Analysis

Slice cultures were inserted into a temperature-controlled chamber (30°C) mounted on an upright microscope (BX53, Olympus Corporation, Canada) and continuously perfused with Tyrode solution containing: 137 mM NaCl, 2.7 mM KCl, 2.5mM CaCl_2_, 2 mM MgCl_2_, 11.6 mM NaHCO_3_, 0.4 mM NaH_2_PO_4_, and 5.6 mM Glucose (pH 7.4). To determine excitability, whole-cell patch clamp recordings were performed in current-clamp mode from CA1 neurons using borosilicate glass pipettes (3-5 MΩ; GC150TC; Clark Instruments, UK). All recordings were made using an Axopatch 200A amplifier (Molecular Devices, Sunnyvale, CA, U.S.A.). Patch pipettes were filled with intracellular solution containing: 120 mM K-Gluconate, 1 mM EGTA, 10 mM HEPES, 5 mM Mg-ATP, 0.5 mM Na-GTP, 5mM NaCl, 5mM KCl, 10mM Phosphocreatine, and pH was adjusted to 7.3 with KOH. Cells that did not survive the entire recording were excluded from the final analysis. Spikes were detected offline and 3 or more spikes were grouped into bursts if the inter-spike interval was smaller than 600 ms.

### Cell Viability Assay with Propidium Iodide

Hippocampal slice cultures were tested for cell death. Briefly, slices were incubated with 5 µg/ml of propidium iodide (PI) (Sigma-Aldrich, St. Louis, MO) and imaged (Axioplane Imaging Fluorescence Microscope, Carl Zeiss MicroImaging GmbH, Hamburg, Germany). Incubation of mouse hippocampus culture-slices in 15.7M KCl for 5 minutes was used to induce cell death in the KCl positive control slices.

### BrdU Assessment of Cell Proliferation

Hippocampal slice cultures were incubated with BrdU 0.5 µM for 3 days. Following the indicated treatments, slices were fixed at in 0.1 M phosphate buffer (PB; pH 7.4) with 4% paraformaldehyde overnight at 4 °C and washed extensively. The slices were then removed from their coverslips for BrdU staining. DNA was denatured using 50% formamide in 2 × SSC for 1.5 hour for 65 °C. After washing in 2 × SSC buffer, they were incubated at 37 °C in 2N HCL. The slices were then washed in 0.1 M PB and permeablized for 2 days at 4 °C in 0.1 M PB, 0.4% Triton X100 and 10% heat-inactivated horse serum. Primary antibodies (sheep a-BrdU, Fitzerald Industries, MA, USA) were applied overnight at 4 °C in 0.1 M PB + 0.4% Triton X-100 and 5% NHS. Secondary antibodies (donkey a-sheep Alexa-568 secondary antibodies; Molecular Probes, OR, USA) were then added overnight at concentration of 1:300. Dentate gyrus region of the hippocampal slices were imaged on an upright confocal microscope (Leica DM6000 B upright microscope; HCX PL APO 63× NA 1.4 oil immersion objective).

### Nuclei Separation from Slice Culture

Nuclei were separated from mouse hippocampus slice using the protocol described by Matevossian et al., 2008 [55] with the following two modification: First, each sample consisted of 5 slices and lyzed in 1ml of lysis buffer; Second, the nuclei were suspended in a final volume of 500 µL. Nuclei were mixed 1:1 with Trypan Blue Stain (Gibco, USA) and quantified on a Neubauer-Improved cell counting chamber (LaborOptic, UK).

### Luciferase cCnstruct Assembly

Rat *gria2* 5’ region and promoter (Figure 6 for physical map) were amplified from 5ng of rat genomic DNA by a PCR reaction using the following primers: Promoter region Forward - TTT GGA TCC GAA GCT AAA GTT CAc agt ttt ggg ag; Reverse - TTT CCA TGG AAT TAG ATC CTC TGC ATT GTG AG; Probe region (sense) Forward - TTT CTG CAG TTC AAG AGC AAT CCA CAG G; Reverse - TTT GGA TCC CTA TGA TGC AAG CAT AAT TCC; Probe region (antisense) Forward - TTT GGA TCC TTC AAG AGC AAT CCA CAG G; Reverse - TTT CTG CAG CTA TGA TGC AAG CAT AAT TCC according to the following cycle: 5min at 95°C followed by 40 cycles of 35 sec at 95°C, 35 sec at 56°C and 70sec/40sec (respectively) at 72°C. Amplification was followed by 10 min extension at 72°C. The PCR product was purified using Quickclean 5M PCR purification kit (Genscript, USA). PCR product containing the 5’ region and pCpGL-Basic plasmid (promoterless Luciferase reporter plasmid lacking any CpGs in its sequence), generously donated by Dr. Rehi’s lab, University hospital, Regensburg, Germany [37]; were digested with PstI and BamHI restriction enzymes (Fermentas International, Canada) in 2xTango buffer, by incubation for 4 hours at 37°C. Digestion products were cleaned using Quickclean 5M PCR purification kit (Genscript, USA). Vector and insert were ligated in a 1:2 molecule number ratio with T4 Ligase (Frementas International, Canada) based on the manufacturer recommendation. Incubation was conducted over-night at 22°C. Plasmids were transformed into PIR-1 competent bacteria (Invitrogen, Life Technologies, Carlsbad, CA) and plated on agar plates with Zeocin (Invitrogen, Life Technologies, Carlsbad, CA) selective antibiotics (25µg/ml). Positive colonies were picked and grown overnight in LB media containing 25µg/ml. Plasmid was extracted using Quickclean 5M Miniprep kit (Genscript, USA) and tested for the insert by digestion with the same restriction enzymes and visualization on a 1.2% agarose gel (1.2 gr agarose in TBE buffer -89mM Tris-Base, 89mM Boric Acid, 2mM EDTA (pH=8.0)). A positive clone was grown in 500ml LB with selective antibiotics (25µg/ml Zeocin) and purified with Qiagen Maxiprep kit, according to the manufacturer manual.

The plasmid containing the 5’ region was *in vitro* methylated using *M.SssI* CpG methyltransferase (New England Biolabs) in a 400µl reaction with the following ingredients: 10µg vector, 2µl SAM, 40µl Buffer, 3µl Enzyme. After 4 hours of incubation, 2µl of SAM and 1µl enzyme were added and the reaction mixture was incubated for additional 4 hours. Mock methylation followed the same protocol with exclusion of the *M.SssI* enzyme. Methylated plasmid was cleaned using Quickclean 5M PCR purification kit (Genscript, USA).

We then inserted an unmethylated PCR amplified fragment containing the *gria2* promoter: Insert and vector were digested with BamHI and NcoI restriction enzymes (Fermentas International, Canada) in 2xTango buffer, by incubation for 4 hours at 37c. Digestion products were cleaned using Quickclean 5M PCR purification kit (Genscript, USA). Vector and the *gria2* promoter insert were ligated in a 1:2 molecule number ratio with T4 Ligase (Frementas International, Canada) based on the manufacturer recommendation. Incubation was conducted over-night at 22 °C. For the promoter plasmid, the promoter region PCR fragment was ligated under the same conditions to an empty pCpGL-Basic plasmid. After inserting the 'unmethylated' DNA, the ligated plasmids were purified from 1.0% agarose gel using Qiaquick Gel Extraction kit resulting in removal of excess unligated unmethylated DNA. The “patch” methylated constructs were transfected directly into SH-Sy5y neuroblastoma cells without cloning in bacterial cells to maintain the state of methylation of the patch. During cloning in bacterial cells the CpG methylation pattern is lost since *E. coli* does not harbor the DNA methyltransferase required to copy CpG methylation during replication.

### Cell Line and Transfection

SH-Sy5y human neuroblastoma cells (Sigma, USA) were grown in F12:DMEM 1:1 media (Gibco, Invitrogen, Life Technologies, Carlsbad, CA) in controlled environment (5% CO_2_, 37 °C). Cells were transfected using X-tremeGene HD (Roche, Germany) according to the manufacturer recommendations. In brief, 100,000 cells were platted on 6 well plates and grown to 80% confluency at the day of transfection. 1µg of vector was mixed with the transfection reagent (1:3 ratio) and added to the cell media. Cells were harvested 48 hour post transfection.

### Luciferase Activity Assay

Cells were washed with PBS (Invitrogen, Life Technologies, Carlsbad, CA) and harvested using a cell scraper (Sarstedt, Germany). Cells were spun down (5min at 1000rpm) and lysed in 30µl lysis buffer (25mM Tris-Phosphate (pH=7.8), 10% Glycerol, 1% Triton-X, 1mg/ml BSA, 2.5mM EDTA (pH=8.0) and 1x Complete-mini EDTA free protein inhibitors (Roch, Germany)) for 5 min on ice. The lysate was spun for 5 min at 13,000 rpm and the supernatant was removed for activity evaluation. 10µl of the lysate was added to each well with 100µl Luciferase assay substrate (Promega, USA). The reactions were read using FluoStar Optime (BMG labtech, Offenburg, Germany) and the results were normalized to the total protein concentration per sample.

The protein concentration was measured using Bradford reaction assay (Biorad, USA) according to the manufacturer recommendations, and read using DU730 UV/Vis spectrophotometer (Beckman Coulter, USA).

### Validation of Upstream Initiation Site

To test whether a 5' transcript is initiated upstream to the known *gria2* TSS we designed 5' forward primers from the 3’ region of the proposed regulatory region (“5’ region”) where a novel transcript would initiate if indeed this promoter is functional in vivo 5'-GGAATTATGCTTGCATCATAG and 3' reverse primer corresponding to the region of the known TSS 5'- AATATCAGCACCCTCCCAT (see physical map in Figure S2), RT-PCR reaction was conducted on DNAse treated (1 µg per 20 µl for 1 h at 37°C) rat hippocampus RNA to exclude the possibility that we amplified genomic DNA rather than RNA. A PCR product was detected using the following cycle: 5 min at 98 °C followed by 46 cycles of 35 sec at 98 °C, 35 sec at 56 °C and 30 sec at 72 °C. Amplification was followed by 10min extension at 72 °C. The PCR was run, visualized andeluted from an E-Gel Clonewell 2% SYBR safe gel (Invitrogen, Israel) using the E-Gel ^®^Agarose electrophoresis system (Invitrogen, Israel). The PCR product was subjected to Sanger sequencing for confirmation of the sequence and its alignment to the DNA upstream of the known TSS using Multialin software [56].

### Statistical Analysis

For the DNA methylation results, we used Mann-Whitney U-test. For mRNA expression analysis, a Student’s t-test compared between treatment groups. To measure correlation between DNA methylation and either bursting or seizures, we first averaged the methylation levels at all 5 CpG sites in the rat *gria2* 5’ region and the 3 CpG sites in the mouse *gria2* 5’ region. The average DNA methylation value per animal was correlated with the number of convulsive seizures experienced, or number of recorded bursts in the slices, using Pearson’s correlation analysis. Significance between the different conditions in multiple-comparisons was calculated using the Student t-test with Holm-Bonferroni correction for multiple comparisons [57]. All analyses were performed using Graph pad Prism or Microsoft Excel software, and results were considered statistically significant when p<0.05.

## Supporting Information

Figure S1 **NormFinder analysis of expression stability of several reference genes.** Variability values calculated by NormFinder for 4 reference genes and a recommended combined reference. Note that all reference genes are below the acceptable variability cutoff previously defined by Wierschke et al. 2010 [31] as v<0.15.
(TIF)

Figure S2 **Upstream *gria2* initiation site.** An RT-PCR reaction was used to validate the existence of an alternate upstream *gria2* TSS *in* *vivo*. (A) Physical map of the rat *gria2* promoter and 5’ region. CpG sites are marked by balloons and numbers indicate distance from previously known TSS. Primers spanning from the 3’ end of the *gria2* 5’ region (red arrow marked F) to the 5’ end of the known TSS (red arrow marked R) were used to amplify DNAse-treated rat hippocampus RNA. (B) PCR product was produced only after reverse-transcription (cDNA) and not when conducting the PCR reaction directly on the DNAse-treated RNA (RT -- ). (C) The PCR product was subjected to Sanger sequencing and aligned to the *gria2* promoter, from the 5’ region to the previously reported TSS (C).
(TIF)

Table S1 **PCR primers.**
(DOCX)
