# Supplementary figures and images for: DNA Methylation Mediates Persistent Epileptiform Activity In Vitro and In Vivo
Source: PLoS One. 2013 Oct 2;8(10):e76299. doi: 10.1371/journal.pone.0076299 (PMC3788713; doi:10.1371/journal.pone.0076299)

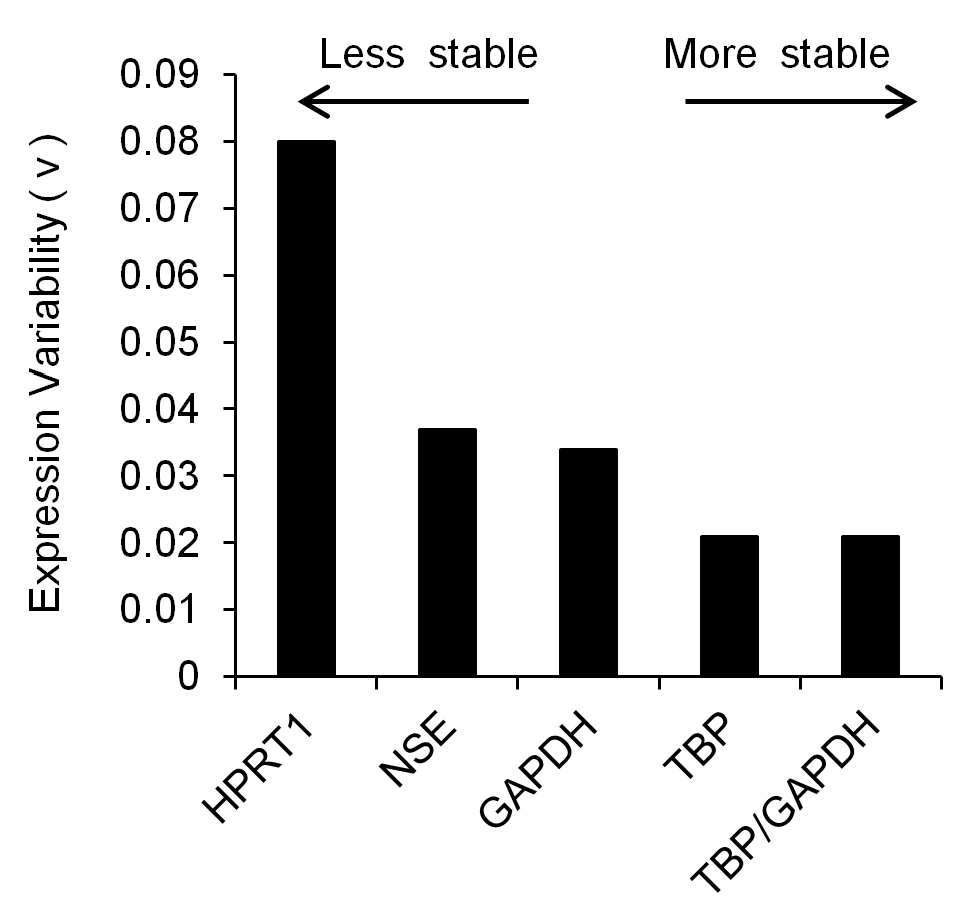

Supplement: Figure S1 — NormFinder analysis of expression stability of several reference genes. Variability values calculated by NormFinder for 4 reference genes and a recommended combined reference. Note that all reference genes are below the acceptable variability cutoff previously defined by Wierschke et al. 2010 [31] as v<0.15. (TIF) [file pone.0076299.s001.tif]

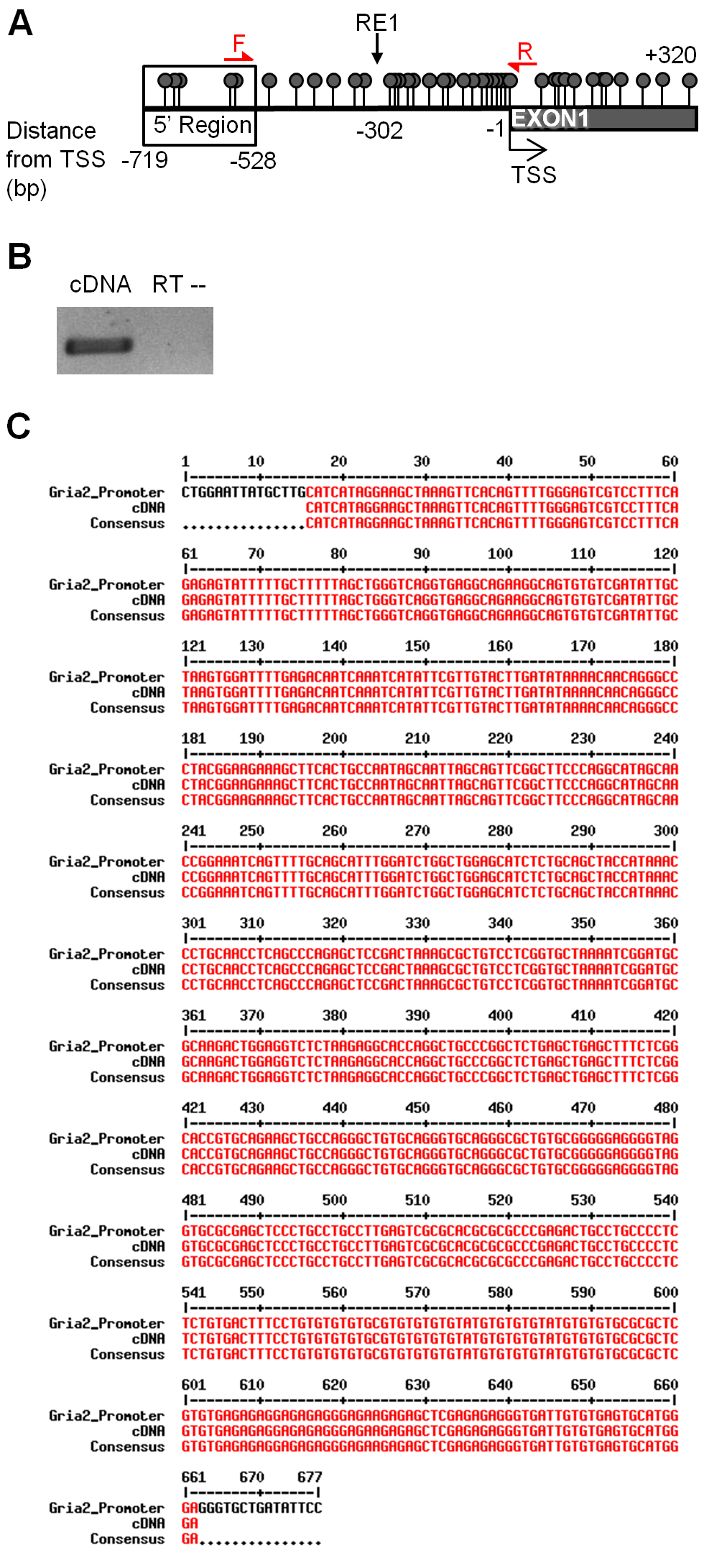

Supplement: Figure S2 — Upstream gria2 initiation site. An RT-PCR reaction was used to validate the existence of an alternate upstream gria2 TSS in vivo. (A) Physical map of the rat gria2 promoter and 5’ region. CpG sites are marked by balloons and numbers indicate distance from previously known TSS. Primers spanning from the 3’ end of the gria2 5’ region (red arrow marked F) to the 5’ end of the known TSS (red arrow marked R) were used to amplify DNAse-treated rat hippocampus RNA. (B) PCR product was produced only after reverse-transcription (cDNA) and not when conducting the PCR reaction directly on the DNAse-treated RNA (RT -- ). (C) The PCR product was subjected to Sanger sequencing and aligned to the gria2 promoter, from the 5’ region to the previously reported TSS (C). (TIF) [file pone.0076299.s002.tif]
