# Supplementary material for: DNA Methylation Mediates Persistent Epileptiform Activity In Vitro and In Vivo
Source: PLoS One. 2013 Oct 2;8(10):e76299. doi: 10.1371/journal.pone.0076299 (PMC3788713; doi:10.1371/journal.pone.0076299)
Supplement: Table S1 — PCR primers. (DOCX) [file pone.0076299.s003.docx]

Table S1: PCR primers

| Bisulfite Sequencing primers | | | | | |
| --- | --- | --- | --- | --- | --- |
| Mouse | Part 1 | Out | Fwd | TAGGAAAGTTATAGAGAGGGGTAGGTA | |
| Gria2 |  |  | Rev | ACCCTACAACCTCAACCCATA | |
| Promoter |  | In | Fwd | GTTATAGAGAGGGGTAGGTAG | |
|  |  |  | Rev | Biot-CTACAACCTCAACCCATAACA | |
|  |  | Seq | 1 | GTATTTAGTATAGTTTTGGTAG | |
|  |  |  | 2 | AGAGGGGTAGGTAGT | |
|  |  |  | 3 | GGTAGGAGGAGAGTTAGAGTATTTA | |
|  | Part 2 | Out | Fwd | GGTTTAGAGTAGGGTAGTTTGGT | |
|  |  |  | Rev | TAAACCCTACAACCTCAACCCAT | |
|  |  | In | Fwd | AGAGTAGGGTAGTTTGGTGTTTT | |
|  |  |  | Rev | Biot-CCTACAACCTCAACCCATAACA | |
|  |  | Seq |  | GTTTGGTGTTTTTTAGAGATT | |
|  | Part 3 | Out | Fwd | TTGAGGTTGTAGGGTTTATTGTAGTT | |
|  |  |  | Rev | AAATTTTAAAACAATCAAATCATATTC | |
|  |  | In | Fwd | Biot-ATTTAAATGTTGTAAAATTGATTTT | |
|  |  |  | Rev | TACTCAATATAAAACAAAACCCTAC | |
|  |  | Seq |  | ATAAAACAAAACCCTAC | |
|  | Part 4 | Out | Fwd | GGGTTTATTGTAGTTGTAGAGA | |
|  |  |  | Rev | AAAAACAATCCACAAACAATATAAC | |
|  |  | In | Fwd | GGGTTTTGTTTTATATTGAGTATA | |
|  |  |  | Rev | Biot-CCACTATCCAAAAAAACATACTCT | |
|  |  | Seq |  | TTTTTAATTATAAAAGGATG | |
|  | 5’ | Out | Fwd | TGTGGATTGTTTTTGTATTATAGT | |
|  | Region |  | Rev | AAACCCCTTCTCACAAACTTTA | |
|  |  | In | Fwd | GGATTGTTTTTGTATTATAGTGTA | |
|  |  |  | Rev | Biot-ACATCCATTCTAAACTACTACC | |
|  |  | Seq |  | GTAGATTTGGTTTTTAGATG | |
| Rat Gria2 | 5’ | Out | Fwd | AAAGTAAAAATATTTTTTGAAAGGA | |
|  | Region |  | Rev | ATCTAAAAACCAAATCTACATAACC | |
|  |  | In | Fwd | TTTATGATGTAAGTATAATTTTAGGGAAAT | |
|  |  |  | Rev | Biot-TTCAAAAACAATCCACAAACAATAC | |
|  |  | Seq | 1 | ATTTTTAATTATAAAAGATGT | |
|  |  |  | 2 | GTTTTTTTGGGTTATGG | |
| *Real-time Quantitative PCR* | | | | | |
| Gria2 | | | Fwd | TGGAGTATTCTACATCCTTGTCGG | |
|  | | | Rev | GCCCTTGACTTGTAACAGAACTCA | |
| GAPDH | | | Fwd | AAATGGTGAAGGTCGGTGTG | |
|  | | | Rev | TGAAGGGGTCGTTGATGG | |
| HPRT1* | | | Fwd | Ctcatggactgattatggacaggac | |
|  | | | Rev | gcaggtcagcaagaacttatagcc | |
| TBP* | | | Fwd | Ccgtgaatcttggctgtaaacttg | |
|  | | | Rev | gttgtccgtggctctcttattctc | |
| NSE | | | Fwd | GGC TTT GCC CCC AAT ATC CT |  |
|  | | | Rev | AAC TCA GAG GCA GCC ACA TC |  |

*HPRT1 and TBP primers sequences were taken from Pernot et al 2010 [[28](#_ENREF_28)]
